# Semen Parameters and Chromatin Packaging in Microsurgical Varicocelectomy Patients

**Published:** 2012-12-17

**Authors:** Marziyeh Tavalaee, Homayon Abbasi, Mohammad Reza Deemeh, Farinaz Fotohi, Mohammad Ali Sadoughi Gilani, Mohammad Hossein Nasr Esfahani

**Affiliations:** 1 Department of Reproduction and Development, Reproductive Biomedicine Research Center, Royan Institute for Biotechnology, ACECR, Isfahan, Iran; 2 Isfahan Fertility and Infertility Center, Isfahan, Iran; 3 Department of Andrology, Reproductive Biomedicine Research Center, Royan Institute for Reproductive Biomedicine, ACECR, Tehran, Iran

**Keywords:** Varicocelectomy, Sperm Parameters, Protamine Deficiency, Pregnancy, Miscarriage

## Abstract

**Background:**

Varicocelectomy is considered as standard treatment for male infertility for clinical varicocele. The aim of this study is to address the effects of varicocelectomy on semen parameters, chromatin packaging, and pregnancy outcome.

**Materials and Methods:**

This retrospective study was carried out between June 2006 and February 2011 on 145 infertile men with grade II or III varicocele. Microsurgical varicocelectomy was performed as part of patient management. Sperm count, motility, morphology, and chromatin packaging were assessed with a Makler counting chamber, light microscopy, Papanicoulaou and chromomycin A3 (CMA3) staining, respectively. In addition, we assessed spontaneous clinical pregnancy and miscarriage rates.

**Results:**

The percentages of spontaneous cumulative pregnancies post-surgery were 33.1% (3 months), 42.06% (6 months), 46.2% (9 months), 48.9% (12 months), and 55.8% (after 12 months). Percentages of spontaneous cumulative miscarriage post-surgery were 2.46% (3 months), 4.93% (6 months), 4.93% (9 months), 6.17% (12 months), and 6.17 % (after 12 months). Both sperm parameters improved and the percentage of sperm protamine deficiency decreased significantly after varicocelectomy.

**Conclusion:**

These results confirm that varicocelectomy improves sperm parameters and chromatin packaging, thereby improving the chance of pregnancy. Positive aspects of this study include the large number of patients studied, duration of follow up, one surgeon who performed all of the surgeries, and type of surgery (microsurgery). The spontaneous pregnancy results also suggest that if pregnancy is not achieved within twelve months post-surgery, an alternative approach such as assisted reproductive technology (ART) treatment should be considered.

## Introduction

Varicocele is an almost symptomless disease that refers to the dilatation and winding of the pampiniform plexus. Varicocele is considered to be a common cause of male infertility and its pathophysiology is not exactly clear (1 ). This condition is associated with high testicular temperature which adversely affects spermatogenesis, thereby reducing semen parameters, sperm quality and function (2 ). A World Health Organization (WHO) multicenter study conducted on 9034 men from 34 centers in 24 countries, reported clinical varicocele in 25.4% of men with changed semen quality and only 11.7% of men with normal semen parameters. Based on such studies, varicocele repair has been suggested and a large number of reports have revealed an improvement in the semen parameters following varicocelectomy (3 ).

It is well documented that infertile men with varicocele present higher levels of sperm DNA damage (4 ). Sperm DNA damage is a multifactorial process possibly induced by external and internal factors such as apoptosis, extreme production of reactive oxygen species, and abnormal chromatin packaging. The replacement of nucleosome histones by protamines during spermiogenesis results in chromatin condensation and sperm maturation. The absence of protamine and/or presence of excessive histones are sign of immature sperm and reduce the sperm's fertilizing capabilities (5 , 6 ).

Despite the aforementioned reports that recommend varicocele repair to improve fertility potential (7 -10 ), there are some critiques of this procedure. Yamamoto et al. have stated "the pregnancy rate in a group treated with varicocelectomy was not statistically higher than that in an untreated group, although subclinical varicocelectomy partially improved seminal parameters" (11 ). This discrepancy might be the result of several factors that include variations in patient populations among studies, methods of assessment, expertise of medical personnel, low patient numbers, and variation in semen parameters within an individual, along with other possible factors. These variations have made meta-analyses difficult for semen parameters, DNA fragmentation or chromatin integrity (10 ).

Therefore, the main objective of this study was to compare semen parameters and chromatin packaging between individuals who benefitted from microsurgical varicocelectomy to those who showed no benefit. This study was conducted over a 12-month follow up in a large number of varicocele patients.

## Materials and Methods

We conducted this retrospective study at Royan Institute and the Isfahan Fertility and Infertility Center. Semen analysis, history, and physical examinations were performed for each participant. Each participant signed an informed consent. The Ethics Committee for Research Involving Human Subjects at Royan Institute approved this study.

### Sample size

The data presented in this study were obtained from 145 individuals with grades II or III varicocele who referred to the Isfahan Fertility and Infertility Center for microsurgical varicocelectomy between June 2006 and February 2011. Semen samples were collected twice by masturbation after 3-4 days of abstinence, once before surgery and once 3 months post-surgery. The samples were analyzed for semen parameters and chromatin packaging status and categorized according to WHO 2010 criteria as follows: i. oliozoospermic (sperm count <39 × 10^6^/ejaculate); ii. asthenozospermic (motility <40); and iii. teratozoospermic (normal morphology <4%) (12 ). The status of semen parameters among study participants was: 3 (2.1%) oligoasthenoteratozoospermic, 21 (14.4%) asthenozospermic, 16 (11.03%) oliozoospermic, 18 (12.41%) oligoasthenospermic, 2 (1.37%) teratozoospermic, 4 (2.75%) asthenoteratozoospermic, and 81 (55.86%) normozoospermic.

### Inclusion criteria

Patients were diagnosed with grades II or III varicocele by palpation and Doppler ultrasound examination. All were married and the duration of infertility was more than one year. Partners of study participants were below 35 years of age, with normal hormonal profiles, regular menstrual cycles, and no signs of female infertility in their medical records.

### Exclusion criteria

Patients with evidence of urogenital infection were excluded.

### Outcome measures

We defined spontaneous clinical pregnancy as visualization of the gestational sac and observation of a viable heartbeat. At that time, we assessed semen parameters and chromatin packaging status. Sperm count was assessed by a Makler counting chamber and motility by light microscope (13 ). Sperm morphology was evaluated using the Papanicolaou staining technique (14 ). For each sample, chromatin packaging status or protamine deficiency was assessed by chromomycin A3 (CMA3) staining (15 ).

### Microsurgical varicocelectomy

This procedure was performed out according to Goldstein et al.(16 ). In brief, following a small incision over the external inguinal ring, we ligated all external and gubernacular veins. With the aid of an operative microscope (Topcon OMS90, Japan) at ×8 magnification, the internal and external spermatic sheaths were opened in order to expose the testicular arteries, lymphatics, and vas deferens with its associated vessels. Veins were distinguished from arteries by their pulsatile behavior after which they were clipped or ligated and divided (16 ). Varicocele patients underwent microsurgical varicocelectomy. Participants were followed 3, 6, 9, 12, and over 12 months following surgery for evaluation of pregnancy status. Measurements of semen parameters and chromatin packaging status were carried out before surgery and 3 months post-surgery.

### Statistical analysis

Statistical analysis was performed with paired and independent sample t tests to compare sperm parameters and chromatin packaging before and after surgery. In addition we compared these parameters between partners of pregnant and nonpregnant females. We determined statistical significance to be p<0.05.

## Results

Out of 145 patients, the partners of 81 patients had successful pregnancies (55.8%), whereas 64 did not become pregnant (44.2%). The pregnancy percentage in partners of nonnormozoospermic individuals with at least one abnormal semen parameter was 48.4% (31 out 64 couples) and 61.72% (50 out of 81) in normozoospermic individuals with normal semen parameters.

To achieve our goal, we compared semen parameters and abnormal chromatin packaging both before and three months post-surgery in the following: i. total population, ii. partners of pregnant and non-pregnant individuals in the total population, iii. partners of normozoospermic individuals, iv. partners of pregnant and nonpregnant individuals in the normozoospermic population, v. non-normozoospermic population, and vi. partners of pregnant and non-pregnant individuals from the non-normozoospermic population.

**Table 1:** Comparison of sperm parameters and chromatin packaging before and after surgery, before surgery in partner of pregnant and non-pregnant groups, and after surgery in partner of pregnant and non-pregnant groups


Total population	Before surgery		After surgery	

**Sperm count (10^6^/ejaculate)**	97.85 ± 9.5 ^a^		170.54 ± 11.32 ^a^	
	P = 110.85 ± 12.5	NP = 85.66 ± 12.25	P = 172.86 ± 14.86	NP = 162.31 ± 17.66
**Sperm motility (%)**	46.91 ± 1.37 b		53.07 ± 1.29 b	
	P = 47.66 ± 1.62	NP = 45.97 ± 2.36	P = 53.83 ± 1.7	NP = 52.09 ± 2.00
**Normal morphology (%)**	15.72 ± 0.85 ^c^		21.63 ± 0.88 ^c^	
	P = 16.02 ± 1.06	NP = 15.04 ± 1.35	P=22.41 ± 1.14	NP = 21.5 ± 1.39
**CMA3 positivity (%)**	53.51 ± 1.32 ^d^		42.20 ± 1.34 ^d^	
	P = 54.85 ± 1.52	NP = 51.87 ± 2.25	P = 41.28 ± 1.62	NP = 43.35 ± 2.22


P; Pregnant and NP; Non-pregnant. Common letters are significantly different at p<0.05.

## Result of sperm parameters and chromatin packaging in the total population

The mean percentages of sperm with normal morphology before surgery was 15.72 ± 0.85 and after surgery it was 21.63 ± 0.88, which was a significant increase (p<0.001). The mean percentages of sperm motility in varicocele individuals before surgery was 46.91 ± 1.37 and after surgery it was 53.07 ± 1.29, which also increased significantly after surgery (p<0.001). The mean sperm count per ejaculate in varicocele individuals before surgery was 97.85 ± 9.51 and after surgery it was 170.54 ± 11.32 which was significantly lower compared with after surgery (p<0.001). The mean percentage of abnormal chromatin packaging or CMA3- positive sperm in varicocele individuals before surgery was 53.51 ± 1.32 and after surgery it was 42.2 ± 1.34, which decreased significantly (p<0.001; Table 1).

**Fig 1:**
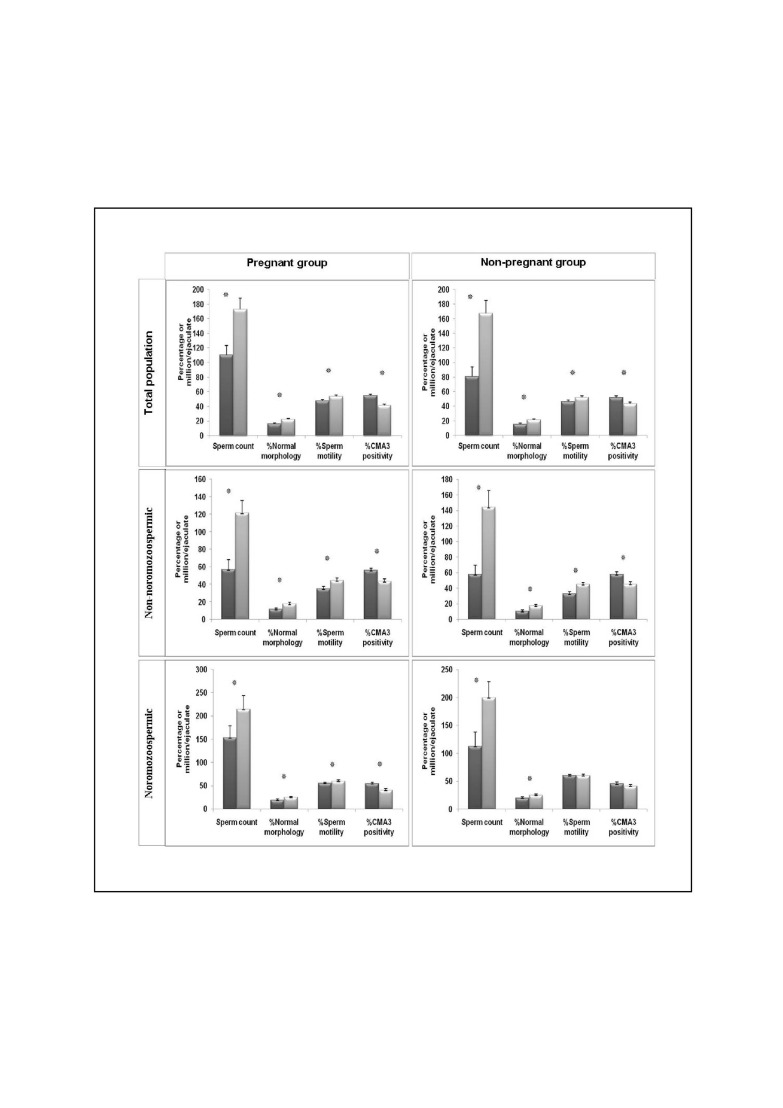
Comparison of sperm parameters and chromatin packaging before and three months following surgery in total population, normozoospermic and non-normozoospermic individuals.

**Table 2:** Comparison of sperm parameters and chromatin packaging before and after surgery, before surgery in partner of pregnant and non-pregnant groups, and after surgery in partner of pregnant and non-pregnant groups of normozoospermic individuals.


Normozoospermic	Before surgery		After surgery	

**Sperm count (10^6^/ejaculate)**	138.91 ± 15.7 ^a^		209.15 ± 17.6 ^a^	
	P = 153.27 ± 19.76	NP = 112.35 ± 25.46	P = 214.30 ± 22.44	NP = 199.62 ± 29.18
**Sperm motility (%)**	57.26 ± 0.99		59.66 ± 1.44	
	P = 55.47 ± 1.08	NP = 60.16 ± 1.83	P = 59.68 ± 1.72	NP = 59.64 ± 2.60
**Normal morphology (%)**	19.22 ± 1.13 ^b^		24.83 ± 1.15 ^b^	
	P = 18.84 ± 1.28	NP = 19.77 ± 2.08	P=24.8 ± 1.43	NP=24.87 ± 1.94
**CMA3 positivity (%)**	50.73 ± 1.69 ^c^		40.51 ± 1.70 ^c^	
	P = 54.18 ± 1.93	NP = 45.29 ± 2.89	P = 40.03 ± 1.97	NP = 41.29 ± 3.15


P; Pregnant and NP; Non-pregnant. Common letters are significantly different at p<0.05.

### Status of sperm parameters and chromatin packaging in partners of pregnant and non-pregnant individuals in total population

In order to evaluate which parameters more profoundly affected pregnancy, we compared both sperm parameters and chromatin status in the partners of pregnant and non-pregnant groups both before and three months after surgery (Fig 1). In the pregnant group, sperm count (110.21 ± 13.28 vs. 172.86 ± 14.86), percentage of sperm with normal morphology (16.02 ± 1.06 vs. 22.05 ± 1.15), motility (47.66 ± 1.62 vs. 53.83 ± 1.70), and CMA3 positive (54.84 ± 1.54 vs. 41.28 ± 1.62) significantly improved compared to before surgery. In the partners of the non-pregnant group, the mean sperm count (80.59 ± 13.00 vs. 167.31 ± 17.66), percentage of sperm with normal morphology (15.33 ± 1.38 vs. 21.11 ± 1.39), motility (45.97 ± 2.36 vs. 52.09 ± 2.00), and CMA3- positive (51.87 ± 2.25 vs. 43.35 ± 2.22) sperm also significantly improved compared to before surgery.

In order to show that the above observations were not due to initial differences between the two groups, we compared sperm parameters and chromatin packaging status before surgery in both pregnant and non-pregnant groups; the results showed that the sperm count (110.85 ± 12.56 vs. 85.66 ± 12.25), percentage of sperm with normal morphology (16.02 ± 1.06 vs. 15.04 ± 1.35), motility (47.66 ± 1.62 vs. 45.97± 2.36), and CMA3- positive (54.85± 1.52 vs. 51.87 ± 2.25) sperm were not significantly different between the pregnant and non-pregnant groups. These parameters were also compared after surgery in both pregnant and non-pregnant groups and were not significantly different (Table 1).

### Result of sperm parameters and chromatin packaging in normozoospermic population

The mean sperm count per ejaculate in varicocele individuals before surgery was 138.91 ± 15.7 and after surgery it was 209.15 ± 17.6, which was significantly (p<0.001) higher compared with before surgery. The mean percentage of sperm motility in varicocele individuals before surgery was 57.26 ± 0.99. After surgery, it was 59.66 ± 1.44, which was not significant (p=0.1). The mean percentages of sperm normal morphology in the before surgery group was 19.22 ± 1.13, following surgery it was 24.83 ± 1.15, which was a significant increase (p<0.001).

The mean percentages of abnormal chromatin packaging or CMA3-positive sperm in varicocele individuals before surgery were 50.73 ± 1.69 and after surgery it was 40.51 ± 1.7. This marker reduced significantly after surgery (p<0.001; Table 2).

### Status of sperm parameters and chromatin packaging in normozoospermia partners of pregnant and non-pregnant individuals

A comparison of semen parameters noted significant improvements following surgery in partners of pregnant individuals compared to before surgery in terms of sperm count (153.27 ± 19.76 vs. 214.3 ± 22.4; p=0.001), percentage of sperm with normal morphology (18.84 ± 1.28 vs. 24.8 ± 1.43; p=0.00), motility (55.47 ± 1.08 vs. 59.68 ± 1.72; p=0.024), and CMA3- positive (54.18 ± 1.93 vs. 40.03 ± 1.97; p=0.00) sperm. As shown in Figure 1, in the partners of the non-pregnant group, with the exception of sperm motility (60.16 ± 1.83 vs. 59.64 ± 2.6; p=0.829), there were significant improvements in CMA3-positive sperm (45.29 ± 2.89 vs. 41.29 ± 3.15; p=0.063), sperm count (112.35 ± 25.46 vs. 199.62 ± 29.18; p=0.007), and percentage of sperm with normal morphology (19.77 ± 2.08 vs. 24.8 ± 1.94; p=0.004).

### Result of sperm parameters and chromatin packaging in the non-normozoospermic population

The mean sperm count per ejaculate in varicocele individuals before surgery was 57.50 ± 7.93, whereas after surgery it was 132.60 ± 12.4 (Table 3), which was significantly higher (p<0.001). The mean percentage of sperm motility in varicocele individuals before surgery was 33.82 ± 1.83 and after surgery it was 44.72 ± 1.82, which also increased significantly (p<0.001). The mean percentage of sperm normal morphology before surgery was 11.15 ± 1.03 and after surgery was and 17.47 ± 1.19. This parameter increased significantly after surgery (p<0.001).

The mean percentages of abnormal chromatin packaging or CMA3-positive sperm in varicocele individuals before surgery were 57.04 ± 2.02. After surgery this percentage reduced to 44.35 ± 2.11, which was significant (p<0.001).

### Status of sperm parameters and chromatin packaging in non-normozoospermic partners of pregnant and non-pregnant individuals

In the partners of the pregnant group, sperm counts (57.11 ± 11.1 vs. 121.74 ± 13.73), percentage of sperm with normal morphology (11.7 ± 1.57 vs. 17.83 ± 1.67), motility (35.08 ± 2.58 vs. 44.4 ± 2.7), and CMA3-positive sperm (55.93 ± 2.57 vs. 43.33 ± 2.83) significantly improved after surgery (p<0.05). Similarly, in the partners of the non-pregnant group, sperm counts (57.91 ± 11.54 vs. 144.24 ± 21.35), percentage of sperm with normal morphology (10.58 ± 1.36 vs. 17.1 ± 1.73), motility (32.63 ± 2.63 vs. 45.00 ± 2.46), and CMA3-positive sperm (58.06 ± 3.1 vs. 45.28 ± 3.14) significantly improved after surgery (p<0.05) in partner of non-normozoospermic individuals (Fig 1).

### Cumulative pregnancies and miscarriage rate

**Fig 2:**
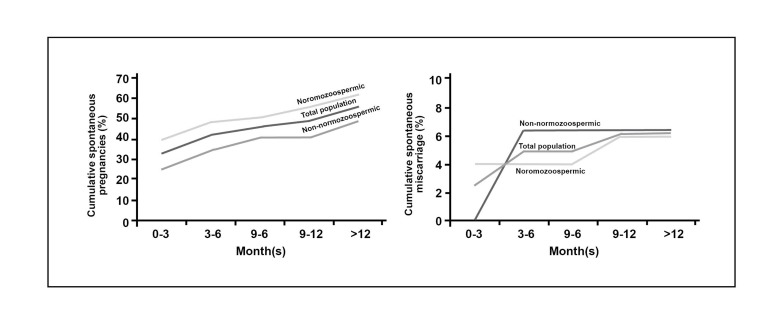
Percentage of cumulative spontaneous pregnancies and miscarriages after 3, 6, 9, 12 and over 12 months after surgery in total population, normozoospermic and non- normozoospermic individuals.

The percentages of cumulative spontaneous pregnancies following surgery in total population were 33.1% (3 months), 42.06% (6 months), 46.2% (9 months), 48.9% (12 months), and 55.8% (over 12 months). Percentages of spontaneous cumulative miscarriages following surgery in total population were 2.46% (3 months), 4.93% (6 months), 4.93% (9 months), 6.17% (12 months), and 6.17% (over 12 months). In addition, percentages of cumulative spontaneous pregnancies and miscarriage separately are shown in figure 2 in the three groups (whole population, normozoospermic, and non-normozoospermic populations).

**Table 3:** Comparison of sperm parameters and chromatin packaging before and after surgery, and before and after surgery in partners of pregnant and non-pregnant groups of non-normozoospermic individuals


Normozoospermic	Before surgery		After surgery	

**Sperm count (10^6^/ejaculate)**	57.50 ± 7.93^a^		132.60 ± 12.4^a^	
	56.24 ± 10.77 (P)	60.77 ± 11.35 (NP)	121.74 ± 13.73 (P)	144.24 ± 21.35 (NP)
**Sperm motility (%)**	33.82 ± 1.83^b^		44.72 ± 1.82^b^	
	35.08 ± 2.58 (P)	32.63 ± 2.63 (NP)	44.41 ± 2.74 (P)	45.00 ± 2.46 (NP)
**Normal morphology (%)**	11.15 ± 1.03^c^		17.47 ± 1.19^c^	
	11.70 ± 1.57 (P)	10.58 ± 1.36 (NP)	17.83 ± 1.67 (P)	17.1 ± 1.73 (NP)
**CMA3-positivity (%)**	57.04 ± 2.02^d^		44.35 ± 2.11^d^	
	55.93 ± 2.57 (P)	58.06 ± 3.10 (NP)	43.33 ± 2.83 (P)	45.28 ± 3.14 (NP)


P; Pregnant and NP; Non-pregnant. Common letters are significantly different at p<0.05.

### Female age, male age, and duration of infertility in pregnant and non-pregnant groups of the total population

Female age, male age, and duration of infertility were compared between the pregnant and nonpregnant group. The female age in the pregnant group was 26.52 ± 4.67 years; in the non-pregnant group it was 27.13 ± 4.71 years, which was not significant. The male age in the pregnant group was 30.7 ± 4.43 years and in the non-pregnant group, it was 31.96 ± 5.48 years, which also was not significant. However, the duration of infertility (year) was 2.6 ± 2.06 in the pregnant group and 3.48 ± 2.32 in the non-pregnant group, which was significant (p<0.05).

### Female age, male age, and duration of infertility in the pregnant and non-pregnant normozoospermic groups

The female age was 26.43 ± 4.82 years in the pregnant group and 26.78 ± 4.84 years in the nonpregnant group. The male age was 30.43 ± 4.03 years in the pregnant group and 31.12 ± 5.55 years in the non-pregnant group of this population, both of which were not significant. However, there was a significant difference (p<0.05) in the duration of infertility (year) between the pregnant group (2.64 ± 1.93) and the non-pregnant group (3.25 ± 2.39).

### Female age, male age, and duration of infertility in the pregnant and non-pregnant non-normozoospermic population

The female age was 26.64 ± 4.55 years in the pregnant group and 27.4 ± 4.68 years in the nonpregnant group of this population. The male age was 31.03 ± 4.94 years in the pregnant group and 32.63 ± 5.42 years in the non-pregnant group of this population, neither of which was significant. However, the duration of infertility (year) was 2.54 ± 2.28 in the pregnant group and 3.81 ± 2.34 in the non-pregnant group, which was significant (p<0.05).

## Discussion

The incidence of varicocele is higher in infertile individuals, yet the underlying cause of the disease is unclear (17 ). Based on the literature, five possible mechanisms may account for varicocele: i. increased testicular temperature, ii. increased adrenal or renal toxic metabolites, iii. testicular oxidative stress, iv. dysfunction of the hypothalamic-gonadal axis, and v. testicular hypoxia induced by venous stasis (1 , 18 ). Despite these proposed mechanisms, the impact of varicocele repair on fertility remains controversial. Two opinions have been stated by Zucchi et al. regarding treatment of infertile men with varicocele, surgery for treatment of varicocele in patients with clinical varicocele and those has primary infertility. A second group of authors have stated their opposition to varicocelectomy and suggest that, despite improved sperm parameters, they observed no beneficial difference in pregnancy rate compared to the control group (19 ). Based on these differences of opinion, researchers have suggested that one should focus on the bio-functional properties of sperm in order to evaluate the outcomes of varicocelectomy rather than the sole evaluation of semen parameters. A meta-analysis study has discerned that the mean value for spontaneous pregnancy rate in varicocele individuals who did not undergo surgery was approximately 16%, with a range of 10% to 23%. In the varicocelectomy group the mean value increased to 38% and ranged from 29% to 60% (10 ).

The pregnancy rate in the current study was within the range of the latter group at 55.8%, which suggested that surgery appeared to improve testicular function in the varicocele individual. Of note, positive points in the current study included: the number of patients, duration of follow up, all surgeries were performed by the same surgeon, and the type of surgery (microsurgery).

In this study, semen parameter and chromatin maturity improved following surgery compared to before surgery in the total population in addition to the partners of both pregnant and non-pregnant groups. This has suggested that the sole improvement in these parameters cannot account for difference in pregnancy in the two groups. Importantly, semen parameters and chromatin status before and after surgery were not different between these groups. Overall these results indicated that other factors possibly accounted for the difference in pregnancy outcomes in the two groups. A literature study suggested that DNA integrity has a paramount impact on pregnancy (6 ). Whether such a difference or other underlying causes account for a successful or unsuccessful pregnancy remains to be evaluated.

Literature studies suggest that the initial semen quality affects the pregnancy outcome post-varicocelectomy (20 ). Therefore, in this study we have divided individuals into two groups, normozospermic and non-normozospermic. The assessment of semen parameters and chromatin status in normozospermic individuals who underwent surgery reveal that, with the exception of sperm motility, all parameters significantly improved after surgery. However, a comparison of these parameters in pregnant and non-pregnant individuals before and after surgery revealed that in the pregnant group, all the parameters significantly improved, while in the non-pregnant group only sperm count and normal morphology significantly improved. Improvement for the other two parameters, sperm motility and CMA3, was insignificant. Whether a lack of improvement of these two parameters possibly accounted for the failed pregnancies in this group remains to be evaluated.

The common mechanism involved in motility and chromatin integrity is the status of disulfide bond in the head and tail, respectively (21 ). These parameters are related to the glutathione level. This level is related to the sperm's antioxidant capacity required to deal with reactive oxygen species, which in turn may affect spermolemma, fertilization and pregnancy outcomes (22 -24 ). Improvement in these two parameters may account for the success of varicocele in the pregnant group. However, this conclusion does not rule out other possible factors.

A similar assessment in non-normozospermic individuals revealed that in both the pregnant and non-pregnant groups, all four assessed parameters increased after surgery. We observed no difference between these two groups before and after surgery, which suggested that other factors might have accounted for the unsuccessful surgeries in the nonpregnant group.

Another factor assessed in this study was the cumulative spontaneous pregnancy and miscarriage rate. The results showed that initial semen parameters of individuals affected the pregnancy outcome, since the cumulative spontaneous pregnancy remained higher in normozospermic compared to non-normozospermic individuals. However, the general rate of miscarriage was lower than a previous reported value in the literature and did not different between the two groups (25 ).

## Conclusion

Despite differences observed in the partners of pregnant and non-pregnant individuals in the normozospermic group, we can propose that other factors such as DNA fragmentation, sperm membrane integrity, the ability to undergo capacitation and acrosome reactions, or factors which may affect these functions (such as ROS and epigenetic factors) may account for the difference between pregnant and nonpregnant groups in normozospermic individuals. This should be assessed in a future study with the intent to determine the most important factors which may account for improved pregnancy rates following microsurgical varicocelectomy.
